# Stable isotope analysis in soil prospection reveals the type of historic land-use under contemporary temperate forests in Europe

**DOI:** 10.1038/s41598-024-63563-1

**Published:** 2024-06-26

**Authors:** Martin P. Janovský, Laszlo Ferenczi, Jakub Trubač, Tomáš Klír

**Affiliations:** 1 Department of Archaeology, Faculty of Arts, Charles University, Nám. Jana Palacha 2, 116 38 Prague, Czechia; 2 Institute of Geochemistry, Mineralogy and Mineral Resources, Faculty of Science, Charles University, Albertov 6, 128 43 Prague, Czechia

**Keywords:** Middle ages, Manuring, Soil geochemistry, δ^13^C and δ^15^N, Environmental social sciences, Environmental impact

## Abstract

The determination of δ^13^C and δ^15^N values is a common method in archaeological isotope analysis—in studying botanical and human remains, dietary practices, and less typically soils (to understand methods of agricultural cultivation, including fertilization). Stable isotope measurements are also commonly used in ecological studies to distinguish different ecosystems and to trace diachronic processes and biogeochemical mechanisms, however, the application of this method in geochemical prospection, for determining historic land-use impact, remains unexplored. The study at hand focuses on a deserted site of a Cistercian manor, dating from the thirteenth to fifteenth centuries. Isotopic measurements of anthropogenically influenced soils have been compared to approximately 400 archaeobotanical, soil, and sediment samples collected globally. The results reveal the potential of isotope measurements in soil to study the impact of past land use as isotope measurements identify specific types of agricultural activities, distinguishing crop production or grazing. δ^13^C and δ^15^N ratios also likely reflect fertilization practices and—in this case—the results indicate the presence of cereal cultivation (C_3_ cycle plants) and fertilization and that the site of the medieval manor was primarily used for grain production rather than animal husbandry.

## Introduction

European landscapes have a very dynamic history; in many regions, land-use patterns changed dramatically in the past. According to current research, the extent of cultivated land increased during the High Middle Age, however, during the Late Middle Age and the Early Modern Period this trend reversed, and the area of forests expanded significantly^1–3^. Medieval monasticism was one of the major forces driving historic landscape transformation during the high medieval period. Since its foundation in the late eleventh century, the Cistercian Order has been renowned for its role in bringing into cultivation often inhospitable, marginal landscapes in different regions of Europe. Present-day landscapes still preserve various traces of their economic activities, to the extent that this impact can be documented today, using landscape archaeological and environmental archaeological methods^4–7^. Although the agricultural expansion during the medieval period and the economic impact of monastic orders (particularly the Cistercian Order) on European agricultural landscapes are well documented in the historical literature, the environmental/ecological consequences have been less thoroughly investigated and are less well understood.

Crop manuring is a very important topic in world archaeology^8^. Medieval farming and manuring practices have been studied traditionally on the basis of historical data and landscape archaeological evidence, the latter being focused predominantly on off-site surveys and mapping ceramic ‘carpets’ (surface scatter of ceramic artefacts)^9–13^. Another source of information on landscape cultivation is geochemistry^14,15^, mostly focusing on phosphate analysis and N isotopes in soils, demonstrating their elevated values in connection to ancient and medieval cultivation. As has been illustrated in the examples of certain archaeological sites^16,17^, such traces remain detectable despite later changes in land-cover (e.g. afforestation). But what if isotope measurements also provide information about distinct local or regional strategies in crop cultivation and animal husbandry?

In the present paper, our objective is to conduct a comparative evaluation of δ^13^C and δ^15^N data obtained from soil samples gathered from our study site, a medieval manor and settlement, which belonged to the estate of the Cistercian Abbey in Plasy, in Western Bohemia, Czechia. The comparative datasets include both soil and cereal grain data which have been previously published (for local data from Czechia, see:^18^; for broader, global-scale data, see:^8,19,20^).

We analyze large scale variations in δ^13^C and δ^15^N (specifically in soils, C_3_ and C_4_ plants) and also present a comparative intrasite study, looking at different variables: N%, C%, C:N, N:P, and P concentration. The latter is known as an anthropogenic element, whose potential source of amplification can be fertilization (manure or plant based), however, this should be interpreted cautiously as noted in the literature^21–26^. Our goal is to study isotope variations in soil to understand the extent of agricultural impact on the study area, and partly to differentiate past land use practices.

This study builds upon the existing geochemical research in the area^15^, factoring in the geomorphology and medieval land use, as indicated by LiDAR data. The combination of these techniques provides a comprehensive understanding of the impact of medieval settlement activities on the geochemical readings and their differing spatial distribution reflect the functional uses of the site and the surrounding landscape (cf.^15,26^).

**Figure 1:**
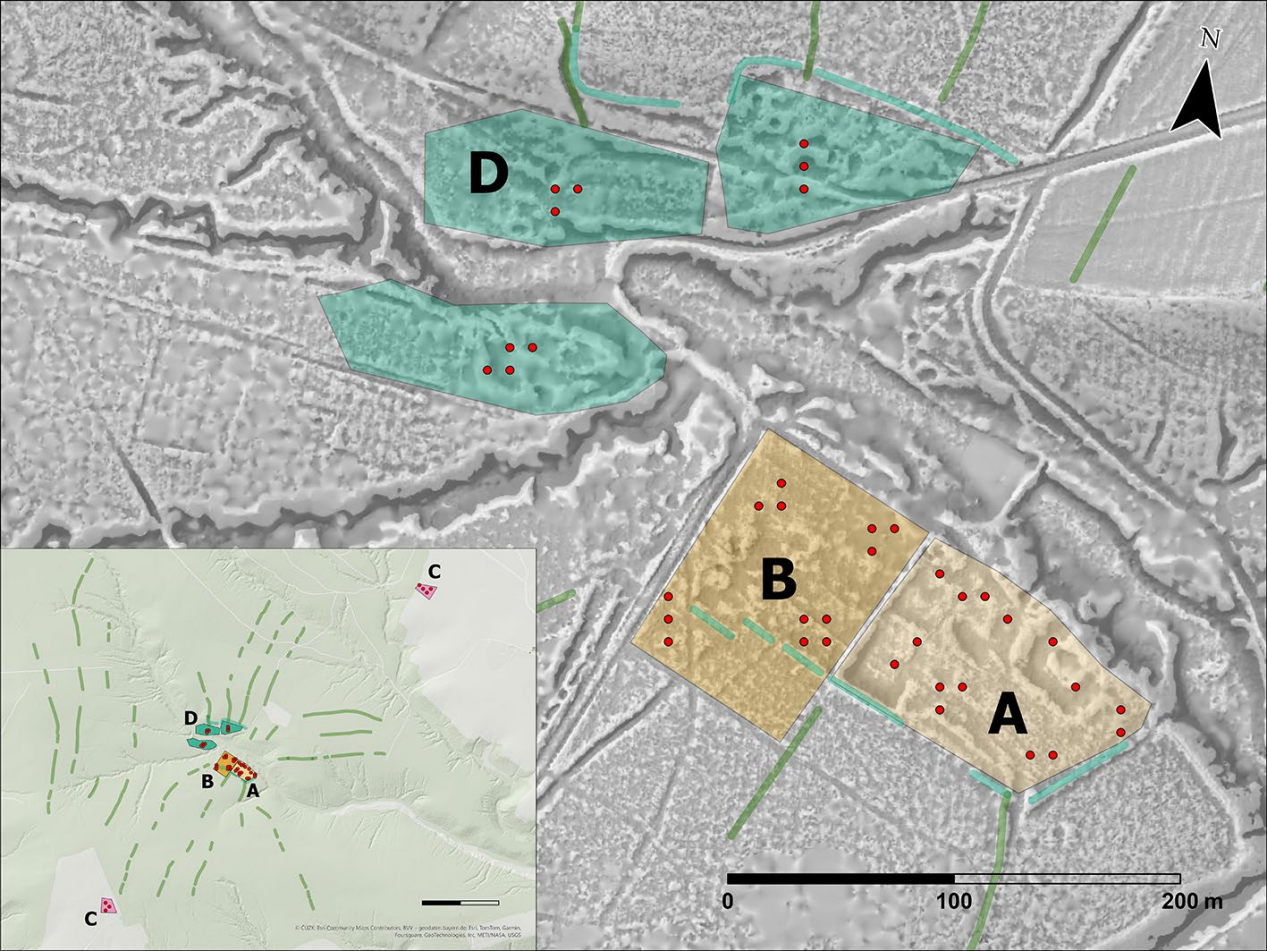
Overview of linear surface features interpreted as field systems based on LiDAR survey in the area of the study site. Zones indicated according to Table 2 (based on LiDAR analysis and interpretation of features by O. Malina): A: ‘COURT’; B: ‘MEDIEVAL FIELD’; C: ‘(MODERN) FIELDS’; D: ‘MEDIEVAL VILLAGE’. Figure was created using ArcGIS Pro 10.3 (https://www.esri.com/en-us/arcgis/products/arcgis-pro/overview), QGIS 3.22.5 (https://www.qgis.org/en/site), and Cloudcompare 2.13 (https://www.cloudcompare.org/main.html). ALS data from ČÚZK Praha (Cadastral Branch Office Prague).

In an innovative approach to reconstruct historical agrarian land use, we aim to associate the aforementioned parameters with observations on historic land use patterns, as documented cartographically and through LiDAR surveys (Fig. 1; interpretation in Table 2). This study highlights two key points. Firstly, soil isotope values reflect the impact of past land use, and they can be used to identify specific types of agrarian use, i.e. differentiate between crop production and grazing. Secondly, the ratios of δ^13^C and δ^15^N in soil bear a likely link to fertilization practices: in this case, they reflect the impact of medieval economic activity in the proximity of the medieval settlement (manorial site): as will be pointed out, the immediate surroundings of the manorial site could be fertilized.

## Results and discussion

**Figure 2:**
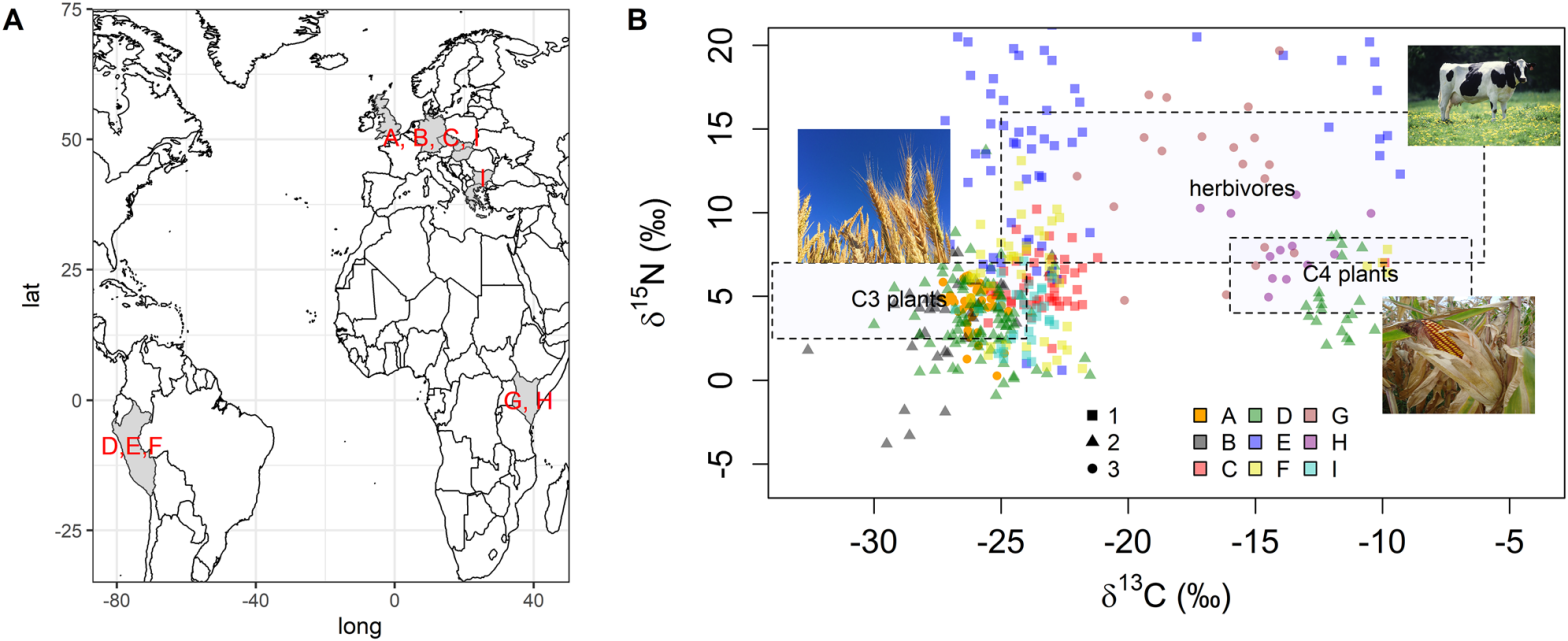
Isotopic binary diagram of site data (**A**) and supporting datasets (**B**–**I**) featured in this study. In the background: boundaries of ecosystems after Staddon^82^. Symbols show three types of data: (1) archaeobotanical samples, (2) modern plants, (3) soil samples (**B**). The map shows in grey the countries from where the datasets were obtained (**A**). Datasets and countries: (**A**–**C**) Czechia and Slovakia, (**D**–**F**) Peru, (**G**, **H**) Kenya, (**I**) Bulgaria, Denmark (Funen island), Germany, Greece, Hungary, United Kingdom.

**Figure 3:**
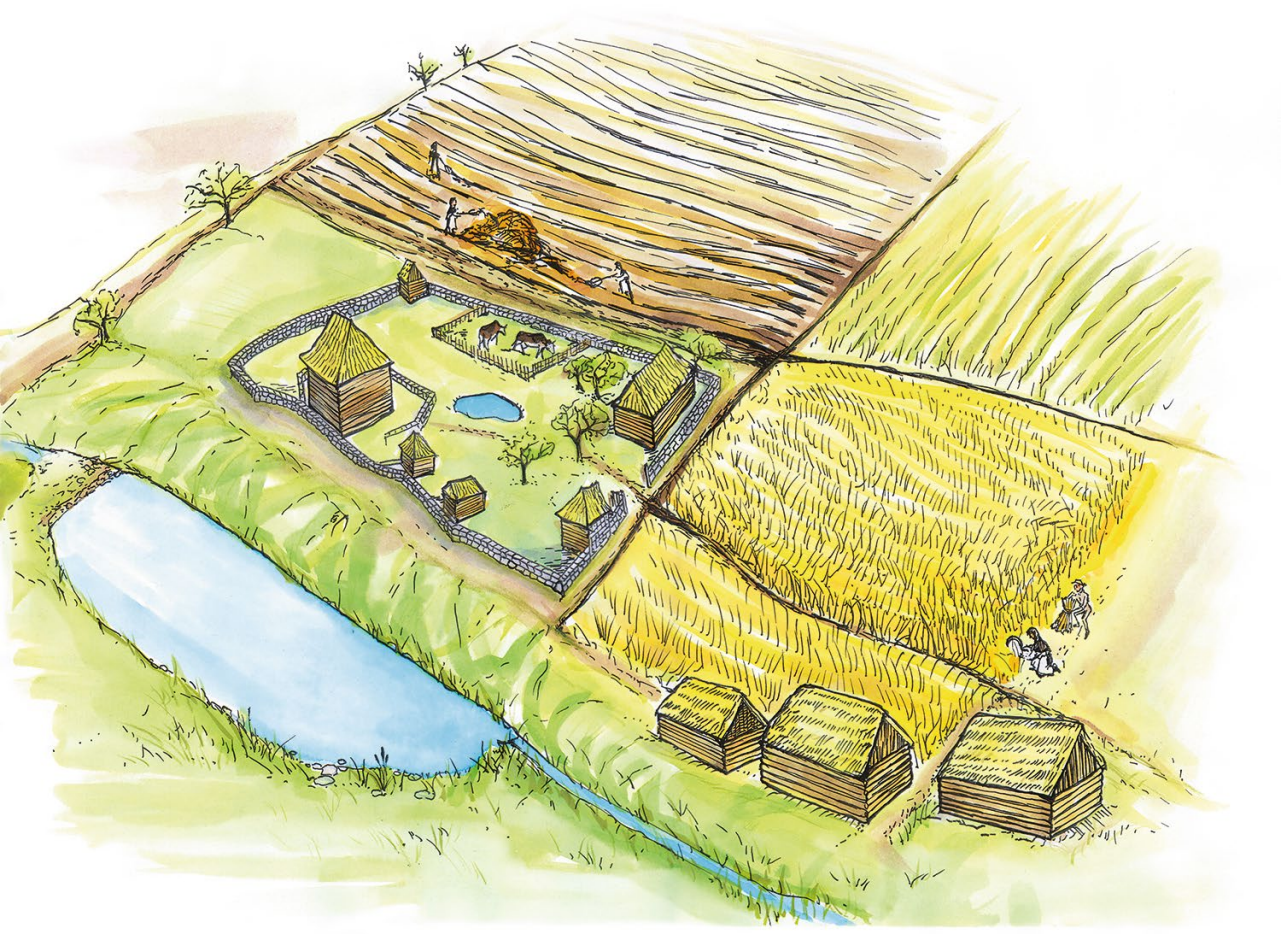
Reconstruction of the Cistercian manor and its surroundings.

Figure 2 shows δ^13^C and δ^15^N values of archaeobotanical, soil and sediment samples divided into 9 groups (A to I according to Table 1, for individual sample results, see: S1). The values measured in soil samples collected from the medieval manor/settlement (A) and its surroundings correspond to the values of plants using the C_3_ cycle. These values are clearly different from those of C_4_ plants and especially of soil and sediment samples associated with pastoral activities of herbivores (G and H). This suggests that the site was not intended for pastoralism, which could be otherwise typical for the Cistercians^6,27^, but that cereals were produced in the area surrounding the court (MEDIEVAL FIELD, Fig. 3). Furthermore, the graph shows that the soils from the precinct of the Cistercian manor and from the area of the adjacent medieval village (COURT, MEDIEVAL VILLAGE), where cereal cultivation was not possible (built-up area), have similar isotope ratios. Apparently, the characteristic building material in the Middle Ages in this region was wood^28^. An important finding of this comparison is, that the values obtained from soil and archaeobotanical remains do not differ. The observed differences are mainly between archaeobotanical samples—uncarbonized with higher δ^15^N (E) and carbonized with lower δ^15^N (F) (for the same observation for C_4_ plants cf.^29^). The difference in δ^15^N between (E) and (F) remains to be explained with regard to contrasting results of previous experimental research on the effect of carbonization^30^. On another note, the samples connected to pastoralism—(G) and (H)—have very distinct manifestations (higher values of δ^15^N and δ^13^C). With regard to the carbon signal, the difference is due to the presence of C_4_ plants in the particular study area (SW Kenya), whose soils were classified as Eutric Cambisols^31^. Dataset (G) consisted of sediments from modern Maasai settlements, contrasting local soil samples used as control^32,33^, which had lower δ^15^N values (4.74, 5.08, and 7.92‰, cf. Figure 2—the points outside the herbivores category zone). Otherwise the values were high, due to cattle and caprine dung.

**Table 1:** Published datasets used in the study.

Code	Data	Type	Samples	Source table	Page	References
A	Trebokov soil samples	Soil samples	45	S1	NA	^15^
B	Modern archaeobotanical samples from Czechia and Slovakia, chaff and grain	Modern plants	38	Table 4a	7	^18^
C	Archaeobotanical samples from Czechia and Slovakia	Archaeobotanical samples	47	Table 4b	8	^18^
D	Modern plants from Peru	Modern plants	110	Table 1	101	^19^
E	Prehistoric samples of plants (uncarbonized) from Peru	Archaeobotanical samples	94	Table 2	102	^19^
F	Prehistoric samples of plants (carbonized) from Peru	Archaeobotanical samples	44	Table 3	104	^19^
G	Modern Maasai setttlements - sediment, soil, dung - pastoral settlements from Kenya	Soil samples	19	Table 1	985	^20^
H	Soil archaeological site (pastoral) from Kenya	Soil samples	12	Table 2	986	^20^
I	Archaeobotanical crop samples from Europe	Archaeobotanical samples	31	Table 2	12592	^8^

**Figure 4:**
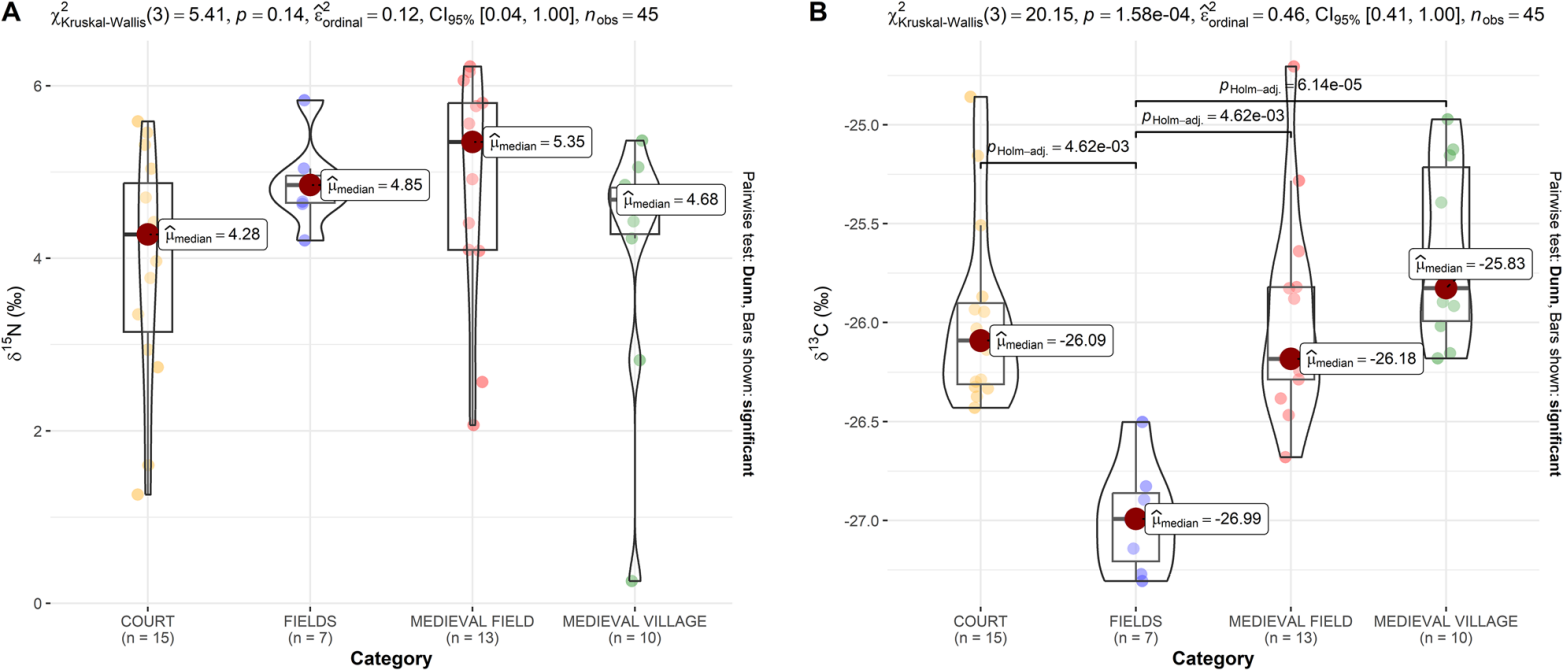
Isotope values of δ^15^N and δ^13^C measured in the area of medieval manor. Categories according to medieval settlement parts and contemporary land use are described in Table 2.

**Figure 5:**
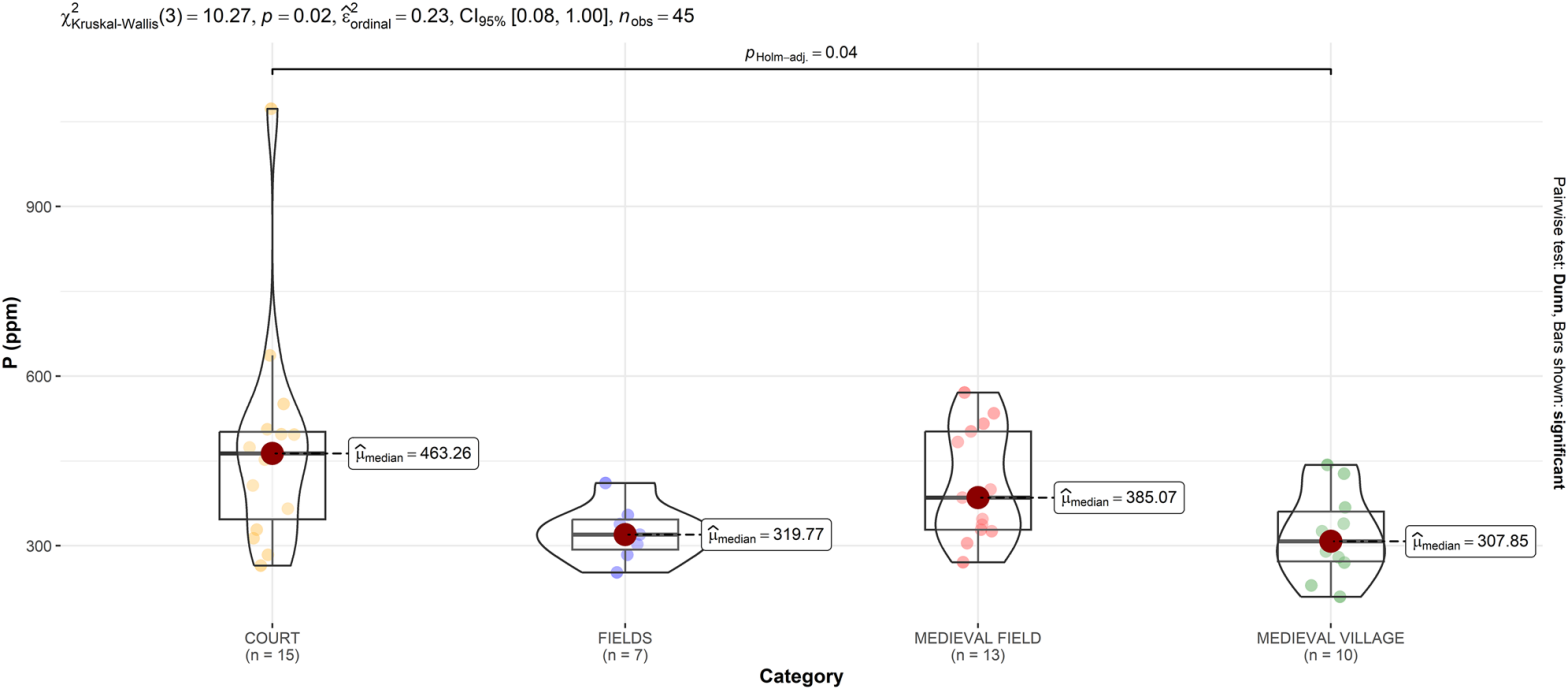
Phosphorus measured in the area of the medieval manor and settlement and in modern fields. Categories according to medieval settlement parts and contemporary land use are described in Table 2.

Secondly, an intrasite comparison of different parameters with regard to different settlement parts and land-use categories (described in Table 2) was carried out. δ^15^N values did not come out as significant (p = 0.144) in the Kruskal–Wallis test (Fig. 4A). The median values are approximately equal, however, they are the highest in MEDIEVAL FIELD, which can be possibly explained by the impact of medieval cultivation and manure/plant based fertilization, as δ^15^N could be an indicator of fertilization and land-use^16,17^. It has been also observed that higher δ^15^N content (fertilization rates) decrease with distance from settlement^34^, and this is also the case at our study site. Phosphorus (Fig. 5) had the highest median value in the COURT area, and its increase is significant there, compared to MEDIEVAL VILLAGE (p = 0.04). This contrast can be associated with archaeological features and sediments, significantly enriched in P, originating from wood ash, household waste and other settlement activity^13,35–37^. Thus, interestingly in our case, δ^15^N appears to be a more suitable indicator of agro-cultivation and fertilization than P, as the concentration of P seems divergent, reflecting the intensity of medieval occupation (Fig. 5).

**Table 2:** Land use categories and number of soil samples from each category.

Category	Current land cover	Samples
COURT	Forest	15
MEDIEVAL FIELD	Forest	13
FIELDS	Ploughland	7
MEDIEVAL VILLAGE	Forest	10

As for δ^13^C values (K–W p < 0.001) in our local context, median values are the highest in MEDIEVAL VILLAGE, relatively high in COURT and MEDIEVAL FIELD, and low in FIELDS (modern fields)—significantly different from all other categories (Fig. 4B). On the one hand, the latter can be linked to the low C content of arable soil, as cereal agriculture can reduce these levels^38^. On the other hand, relatively higher soil carbon levels are generally expected in human settlements and cultivated areas. Anthropogenic soils in these places can be compared to Dark Earth soils^39^. As has been demonstrated in cereal grain and pulse^40^, δ^13^C values remained unaffected by manuring, in contrast to δ^15^N. The variations of δ^13^C have been explained rather in connection to plant variety and climate related factors (more specifically water availability)^41–46^. Such impacts were demonstrated also in paleosoils^47^, however, studies focusing on the effect of manuring in soils found minimal impact on δ^13^C, or inconclusive trends—both positive and negative (see^48^, with further references on^46,49–51^) Furthermore, variations in δ^13^C may be connected to land-cover/land-use patterns and diachronically to vegetation change (deforestation and afforestation). Deforestation could imply C_3_ to C_4_ change, even in this region, in certain archaeological periods^52,53^, however, it should not be factored in this context. Contrastingly, successive afforestation (with pine forest plantations) after the abandonment of the site, and litterfall could potentially impact δ^13^C values in the area (cf.^54,55^).

**Figure 6:**
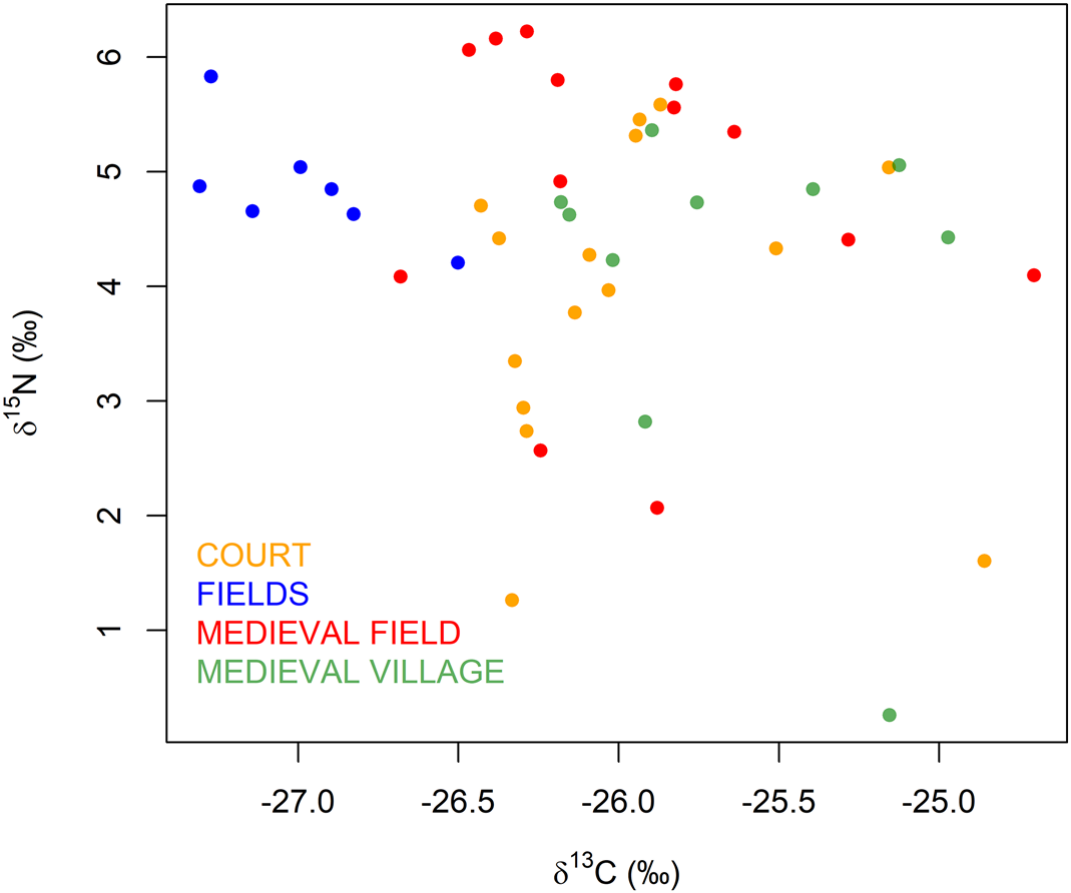
Isotopic binary diagram of values in different parts of the manor and settlement and in modern fields. Categories according to medieval settlement parts and contemporary land use are described in Table 2.

From a comparative perspective, the binary plot of δ^13^C and δ^15^N values (Fig. 6) summarizes similarities and differences between and within the different spatial units in the medieval settlement and its surrounding, and shows the divergence and convergence of individual measurements. In FIELDS, there are converging δ^13^C and δ^15^N values. As stated above, δ^13^C values are characteristically low here, and there are also higher δ ^15^N values here, compared to the highest δ^15^N values in MEDIEVAL FIELD. The δ^15^N and δ^13^C values in MEDIEVAL FIELD, MEDIEVAL VILLAGE and COURT roughly overlap and tend to be more divergent. Generally, the higher values of δ^13^C can be explained in COURT and MEDIEVAL VILLAGE by the decayed medieval buildings that contained carbon, and the higher values in δ^15^N by the accumulation of residential waste in certain locations.

**Figure 7:**
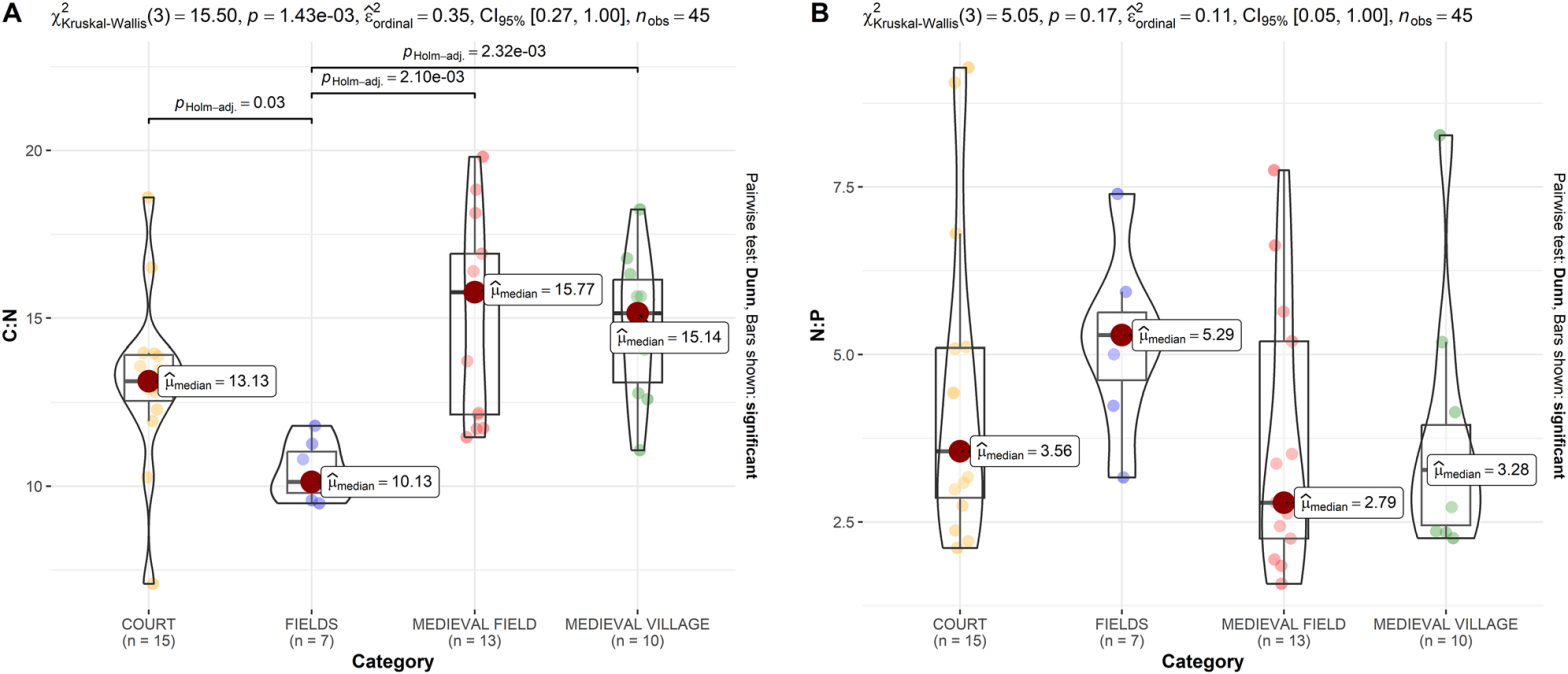
C:N (**A**) and N:P (**B**) ratios measured in different parts of the medieval site and in modern fields. Categories according to medieval settlement parts and contemporary land use are described in Table 2.

Median C:N values (Fig. 7A) are the lowest in FIELDS, and the highest in MEDIEVAL FIELD (K–W p = 0.001). The other two categories associated with the medieval settlement are also higher than FIELDS and significantly different. How well the difference in land use history is reflected by these values is an intriguing question. Given the steady state of land use in the area of the site in the past 200 years at least, and probably even longer, one may hypothetically link back the observed values directly to the differences in medieval land use. Studies analyzing this parameter in formerly uncultivated (forest) soils converted to agricultural land have found decreasing C:N ratios and soil degradation. Compared to agricultural land (fallow, arable), the higher C:N ratios were observed in forest soils (cf.^56^), and was showing a decreasing tendency (in contrast to increased δ^15^N) due to increased land use intensity, or manuring^16^. The impact of agriculturally cultivated land to forest conversion is the opposite (C and N accumulation, with N lagging behind, thus, with increasing C:N)^57,58^ As shown by the comparative investigations of forest tracks, including formerly cultivated ones^59^, the impact of former cultivation could be detected by observing relatively lower C:N ratios. Similarly, one would expect to find relatively lower ratios in MEDIEVAL FIELDS, compared to other forested parts around (not affected by previous cultivation), however, this difference remains unclear in regard to other land use categories documented at this site, which are within the medieval settlement itself, and whose C:N values could all characterize formerly cultivated forest tracks, contrasting FIELDS. In connection to C:N values it is also important to note that traditionally cultivated agro-systems could be generally characterized by stoichiometric homeostasis^60^. In contrast to the use of modern synthetic fertilizers, the use of organic fertilizers maintained or increased both C and N stocks^61,62^. In addition to fertilization, tillage techniques should be also factored in, as demonstrated by C:N decrease in case of traditional tillage compared to minimal disturbance in conservation tillage^63^. In sum, focusing on the C:N ratio alone, it may be difficult to argue for the impact of former cultivation/fertilization and this parameter may not always be an entirely accurate indicator. Without other types of evidence, we might not be able to decide clearly whether differences in C:N ratio between the MEDIEVAL FIELD, the direct vicinity of the manor (COURT) and the adjacent rural settlement (MEDIEVAL VILLAGE) originate from medieval agricultural activity. This is, however, more clearly highlighted by the higher δ^15^N, P, and low N:P values (see the latter in Fig. 7B) found in MEDIEVAL FIELD (K–W p = 0.168).

**Figure 8:**
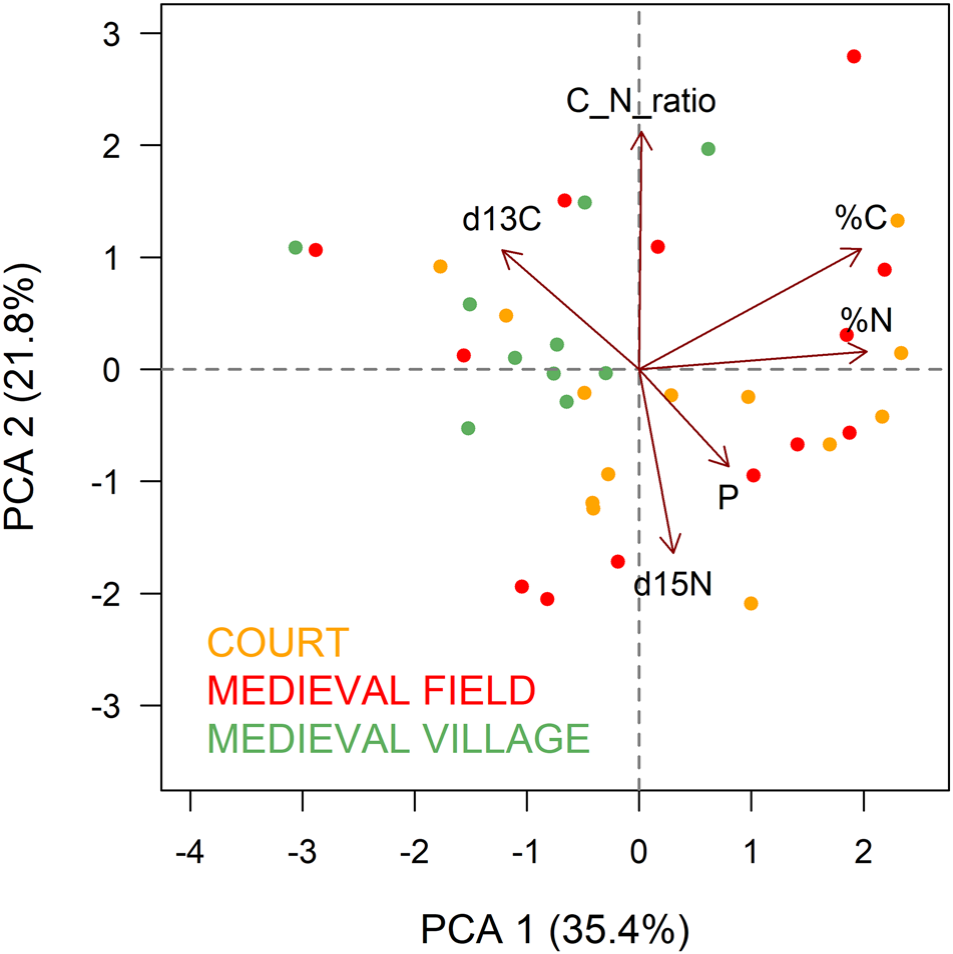
Ordination diagram showing results of PCA analysis comparing different parts of the medieval site. Categories according to medieval settlement parts and contemporary land use are described in Table 2.

Finally, the relation of different parameters in different areas can be demonstrated using PCA (Fig. 8, Table 3). COURT and MEDIEVAL FIELD are associated with %N, %C, δ^15^N, P. MEDIEVAL VILLAGE is associated with δ^13^C. This can be explained by the fact that enrichment within the COURT is mainly due to the presence of anthropogenic features, while in MEDIEVAL FIELD it is most probably linked to cultivation and fertilization.

**Table 3:** PCA results for first two components explaining 57% of variability.

	PC1	PC2
δ^13^C	− 0.38	0.33
δ^15^N	0.10	− 0.51
%N	0.63	0.05
%C	0.62	0.34
C:N	0.01	0.66
P	0.25	− 0.27
Eigenvalue	2.13	1.31
Standard deviation	1.458	1.143
Proportion of Variance	0.354	0.218
Cumulative Proportion	0.354	0.572

In sum, the results of our exploratory study suggest that soil isotope analysis has the potential to identify not only waste dumping near settlement cores, but—using a group of parameters and systematic surveys—it can detect soil signatures of agricultural cultivation and fertilization in archaeological soils. The measured δ^15^N values reflect fertilization, and the comparison of δ^13^C and δ^15^N allows to determine the type of crops grown (C_3_–C_4_), or the impact of grazing. In our case, it appears that the focus of economic activity was cereal production (C_3_ cycle) and not animal husbandry, and this adds another layer to the archaeological interpretation of the site^15^.

In a methodological perspective, stable isotope techniques based on δ^15^N and δ^13^C ratios are widely used in soil ecological studies to distinguish different ecosystems/ecosystem components, to trace and evaluate diachronic processes, biogeochemical mechanisms with special emphasis on food chains and cycling processes (capture, fixation, fractionation, sequestration) affecting soils^64,65^. A common characteristic of soil ecology is the focus on regional, continental and global scales, in order to identify controls along different environmental gradients, i.e. spatial variations in climate, geology, and other factors (to mention a few: Refs.^66–69^ on climate factors (temperature, precipitation); Ref.^70^ on altitudinal differences; Ref.^71^ on global relative abundances of C_3_–C_4_ plants; Refs.^72,73^ on land use gradients-land-use types; Ref.^74^ on local topography; Ref.^75^ on the effects of mycorrhizal fungi; in connection to diachronic seasonal or land use changes). Another line of application is tracking temporal isotope variations in response to short- or long-term diachronic changes (e.g. seasonality, permanent land use change, see^76,77^). Consequently, such studies have already exploited the potential of isotope techniques in identifying/characterizing impacts of past land use on soil conditions^72^. Similarly, the significance of isotope research has been growing in archaeology (particularly in archaeobotany in connection to the theme of agricultural cultivation and farming techniques) and this has led to more robust data collection, to a better understanding of isoscapes, to the production of experimental evidence linking isotope measurements in plant remains and soil^30,78^, and to methods of detecting soil amelioration^79^. Despite these trends, soil geochemical research in archaeology has hitherto given little attention to studying soil isotopes as evidence for past land use in archaeological context. Our paper advances research in that way, advocating the use of such techniques in combination with other parameters and remote sensing data.

## Conclusion

Tracing land-use history in the past is traditionally based on the study of cartographical and archival sources. Regarding the period before the production of spatially accurate information (available only for the past couple of hundred years in the form of maps) one must rely predominantly on textual sources. In the past decades, the application of remote sensing methods and LiDAR in landscape archaeology has improved this research, allowing detailed reconstructions of past land-use changes particularly in connection to the dynamic expansion of agricultural cultivation, documenting relict surface features in afforested areas. Apparently, this type of evidence also has its own limitations, defined by the land-cover and certain types of terrains. The present study puts forward a more generally applicable method, with a potential to contribute to addressing important issues in contemporary economic, social, and environmental history, such as the agrarian crises (of the late Middle Ages), and the mineral exhaustion of agricultural soils.

The present study is an innovative approach to reconstructing historical agricultural land use, using stable isotope analysis of carbon (δ^13^C) and nitrogen (δ^15^N) in soil samples. It demonstrates the possibility to tell from soil isotope compositions whether C_3_ cycle plants were cultivated and used at archaeological sites, which have been abandoned hundreds of years ago. Furthermore, it also illustrates that the impact of agricultural cultivation / fertilization and the C_3_ cycle can be clearly distinguished from pastoral use, which produces scales of δ^13^C and δ^15^N comparatively so different, that it can certainly be rejected as an option at this site. (On the isotope characterization of dung vs manure, see:^80,81^). Our comparative approach also allowed to establish—as a novel result—that soil isotope compositions observed at archaeological sites may not only predict crop cultivation, but also contribute to the assessment of fertilization (in addition to isotopic investigation of archaeobotanical remains). This provides an interesting opportunity for further comparative research at a wider range of archaeological sites. Our comparative datasets illustrate generic physical limits^82^ that are influenced by the C_3_ or C_4_ cycle, or the activities of herbivores, and some other differences—such as between carbonized and uncarbonized samples. Focusing specifically on the selected archaeological site—medieval manor/settlement—we found that its surroundings were not used for grazing, but for grain production. It is also likely that the area in the vicinity of the manor (MEDIEVAL FIELD) was fertilized, which just goes to show how organic waste was managed in the past and distributed in the fields near the settlements. We speculate that not only anthropogenic soils^83^, but any soils on which crops were produced in the distant past carry the above observed signals in the form of altered δ^15^N and δ^13^C. This research opens up new opportunities for the study of historical agricultural strategies and the impact of human activity on the land in an archaeological context. Thus, the analysis of stable isotopes in soil can be a valuable tool for understanding past land use and its long-term consequences.

## Materials and methods

### Study area and site

The area of the Cistercian manor/settlement is geologically homogeneous consisting mainly of Proterozoic consolidated sediments (siltstone, shale and graywacke). The land cover consists of Cambisols^84^ with units classified as dystric and gleyic^15^.

The natural state of the landscape is influenced by previous intrusive settlement, to the extent that anthropogenic soils are present^15,26,83^. The site and its surroundings were settled and used for agriculture from the thirteenth to the fifteenth century. In the early sixteenth century, the settlement was abandoned, and the surrounding landscape was reforested. At least for the last 200 years, the site has been stable in land use, as this is evidenced cartographically^15^. Based on previous research and written records, the study site consists of a manor (COURT), adjacent area of medieval cultivation (MEDIEVAL FIELD), and a nearby medieval village (MEDIEVAL VILLAGE)^15^. The modern agricultural fields (FIELDS) are directly adjacent to the forest boundary (Table 2).

The investigation of LiDAR images revealed traces of medieval cultivation, outlining the extent of village fields. This was confirmed also by geochemical anomalies. Multivariate analysis of geochemical results pointed to diverse economic activities in certain locations^15^. Therefore, these areas have been sampled by hand auger coring. Only the upper B horizon was sampled to obtain information on δ^13^C and δ^15^N in soil. The above-mentioned spatial categories and changes of land use over time are described in Table 2.

### Soil elemental composition analysis

The soil samples were dried at 40 °C for 24 h and sieved to < 2 mm grain size fraction. The fraction was then homogenized (crushed and grinded) in a porcelain mortar. The laboratory analysis using the ED-XRF analyzer (Delta Professional) was based on the methodology which was described in detail^15^. Each measurement took an interval of 1 min–30 s with the 10 kV beam and 30 s with the 40 kV beam.

A comparative ICP-MS measurement was performed on a subset of samples. The analytical processing followed the methodology described by Praus et al.^85^: the pseudo-total elements under investigation were determined in 500 mg of soil, exposed to microwave-assisted wet digestion (Discover SD-D, CEM Corp., USA) at 180 °C for 18 min, using 2 mL HNO_3_ and 6 mL HCl (both Analpure®, Analytika, Czechia). Then, the digested samples were diluted in high-purity water (≥ 18.2 MΩ cm^−1^; Milli-Q purification system, Millipore, SAS, France) and analyzed by inductively coupled plasma mass spectrometry (ICP-MS; Agilent 7700x, Agilent Technologies Inc., US). Measurements were taken several times, averaged, and their standard deviation was computed. The last step was to verify the accuracy of both measurements by comparing them with each other. This comparison resulted in a high correlation for most of the anthropogenic elements. For the Phosphorus used in this study, the Pearson correlation on log-transformed data was 0.9 with p < 0.001.

### Isotope analysis

Stable isotopic composition of nitrogen and carbon is determined using a Thermo Flash 2000 elemental analyser connected to a Thermo Delta V Advantage isotope ratio mass spectrometer in a Continuous Flow IV system. Minimal weight of samples depends on the wt% amount of both elements. Samples wrapped in tin capsules are combusted. Released gases (CO_2_, N_2_) split in a GC column are transferred to MS source through a capillary. Isotope ratios are reported as delta (δ) values and expressed relative to V-PDB scale for δ^13^C and to atmospheric nitrogen for δ^15^N. The raw δ^13^C, δ^15^N and total gas composition values are normalized to the scale using a multiple-point linear regression based on certified international reference materials (e.g., IAEA, Elemental Microanalysis) run during the same sequence. The random error precision is < 2% and measurement error (accuracy) is < 0.1.

### Statistical analysis and datasets

In order to compare the zonation categories of the medieval manor/settlement (Table 2), the non-parametric Kruskal–Wallis test was used^86^. The results of the test with p-values were provided in every Figure. Multiple pairwise comparisons were done using Dunn's test for the groups that are statistically different. Significantly different boxplots are connected by a line. Before performing PCA, the data were scaled and centered. Two outliers detected by library *factoextra* were removed before performing PCA^87^. All analyses were performed in R version 4.1.2 (2021-11-01). To confirm the assumption that the soil preserves site-specific isotope information about medieval land use and fertilization, support datasets were used.

Support datasets:

Comparative datasets of δ^15^N and δ^13^C were used to compare the soil data obtained from the medieval manor/settlement (A) and its surroundings (Table 1, Fig. 2). The first dataset consists of samples of modern plants grown on archaeological soils (B)^18^, and archaeobotanical samples from archaeological sites in Czechia (C)^18^. The second dataset consists of data from another comprehensive study^19^, which consisted of three types of data from Peruvian archaeological sites: samples of modern plants (D), uncarbonized archaeobotanical samples (E), carbonized archaeobotanical samples (F). The third dataset^20^ represents data related to pastoral land use, which partly consists of modern reference sediments from the Maasai settlements (G), as well as archaeological sediments from a prehistoric pastoral settlement (H). The last dataset (I) consists of archaeobotanical samples from European Neolithic sites^8^. A total of 395 samples of both C_3_, C_4_ and legume plant and land use (pastoral) specific data were obtained. To these, soil data from the medieval site (A) were added (45 samples).

### Supplementary Information

Supplementary Information.

## Data Availability

Data are available through our GitHub repository https://github.com/Barilac/SR/.
